# Evaluation of the RDW Index (Red Cell Distribution Width) in Women with Breast Cancer Treated with Doxorubicin in a One-Year Follow-Up Study

**DOI:** 10.3390/diagnostics13091552

**Published:** 2023-04-26

**Authors:** Ricardo Simões, Amanda Cambraia Ferreira, Luciana Maria Silva, Adriano de Paula Sabino, Maria das Graças Carvalho, Karina Braga Gomes

**Affiliations:** 1 Department of Internal Medicine, Faculty of Medical Sciences of Minas Gerais, Belo Horizonte 30130-100, MG, Brazil; 2 Department of Clinical and Toxicological Analysis, Faculty of Pharmacy, Federal University of Minas Gerais, Belo Horizonte 31270-901, MG, Brazil; 3 Research and Development Department, Ezequiel Dias Foundation, Belo Horizonte 30130-110, MG, Brazil

**Keywords:** red cell distribution width, breast cancer, doxorubicin, biomarker

## Abstract

Breast cancer is the most common cancer and the most frequent cause of death in women. Doxorubicin, an anthracycline, is an important drug due to its efficacy in treating solid cancers, especially breast cancer. However, this drug is often responsible for cardiotoxicity that may affect more than 25% of patients. This study aimed to evaluate the red cell distribution width (RDW) in women with breast cancer to monitor adverse events associated with the use of doxorubicin. A prospective study of 80 women with breast malignancy undergoing neoadjuvant doxorubicin-based chemotherapy was conducted. The patients were evaluated at baseline (T0), just after the last cycle of chemotherapy with doxorubicin (T1), and 1 year after the treatment (T2). There was a significant increase over the time points for the RDW (*p* < 0.001). There was a negative correlation between the RDW and C-reactive protein (CRP) levels at T1. The RDW did not show a significant difference between the groups classified according to cardiotoxicity. Based on these results, the RDW is a cost-effective test that shows a relationship with the doxorubicin response, but not with cardiotoxicity. It is a potential biomarker to evaluate patients with breast cancer after they receive chemotherapy with doxorubicin.

## 1. Introduction

The American Cancer Society (ACS) estimated 1,898,160 new cases of and 608,570 deaths from cancer in the USA in 2021 [1]. In this population, female breast cancer represents an estimated 281,550 new cases and 43,600 deaths. Breast cancer is the second-most common type of cancer in the world and the most-common among women, except in cases of non-melanoma skin cancer. Despite being considered a cancer with a good prognosis if diagnosed and treated early, mortality rates remain high in developing countries, most likely because the disease is still diagnosed in advanced stages [2].

Doxorubicin, an anthracycline, is an important drug due to its efficacy in treating solid cancers, especially breast cancer. However, this drug is often responsible for cardiotoxicity that may affect more than 25% of patients. This occurs mainly in the first year after chemotherapy and leads to mortality in 1–4% of patients [3]. In addition to the early diagnosis of breast cancer being a critical point, the application of biomarkers to evaluate the wide spectrum of the therapeutic response and adverse events is necessary. In this way, the use of a simple, automated, and easy-to-obtain test, such as a blood count, can be explored to provide an accurate interpretation of potentially-important data during the follow-ups with patients undergoing treatment.

One of these markers is the variation in the size of red cells—the red cell distribution width (RDW)—a laboratory parameter widely used to quantify anisocytosis, which reflects the variability in the size of circulating erythrocytes [4]. It is reported by automated systems. The RDW is also an early marker of iron deficiency anemia, impaired iron mobilization, and increased oxidative stress. Its main clinical application has been limited, until recently, to the diagnosis of thalassemia trait anemia, as well as an exclusion marker for iron deficiency anemia in cases where serum ferritin does not accurately indicate a disorder in the total iron reserve [5,6]. However, RDW oscillations have been related to many pathophysiological conditions, where its elevation is associated with ischemic heart disease, acute and chronic heart failure, atherosclerosis, and other conditions that evolve with a progressive inflammatory state [7,8,9,10]. The molecular basis of the aforementioned associations has mainly been attributed to the ability of the RDW to reliably reflect increased levels of circulating cytokines, such as interleukin-6 (IL-6) and tumor necrosis factor-alpha (TNF-α) [11].

Huang [12] demonstrated that the RDW was significantly related to breast cancer in young women, and was positively associated with tumor size and the presence of sentinel lymph nodes in samples from the evaluated patients. Another study, which included 104 patients with breast cancer, 100 patients with breast hyperplasia, and 100 healthy patients (control), showed that the RDW increased in patients with breast cancer compared to patients with hyperplasia [13]. Yoo et al. [14] observed that patients with breast cancer with a preoperative RDW over 13.5% had a 1.7-fold higher risk of recurrence and mortality. Moreover, the RDW was correlated with breast parenchymal pattern density from mammography in women with breast cancer [15].

Although cancer is currently considered a disease associated with chronic inflammation [16,17], an increase in the RDW has not been investigated as a potential biomarker in the diagnosis or prognosis of the disease. As far as we know, researchers have not yet evaluated the RDW in patients with breast cancer after treatment with doxorubicin classified according to cardiotoxicity. Therefore, we evaluated the RDW in women with breast cancer treated with doxorubicin in a 1-year follow up study. In addition, we determined the correlation between the RDW and C-reactive protein (CRP), an inflammatory marker.

## 2. Materials and Methods

### 2.1. Subjects

This study was approved by the UFMG Research Ethics Committee (CAAE: 38538714.2.0000.5149), according to the World Medical Association Declaration of Helsinki. Written informed consent was obtained from all participants before inclusion in this study.

The inclusion criteria were as follows: patients aged over 18 years, female, and with breast cancer indicated for neoadjuvant chemotherapy (CT) based on anthracyclines (doxorubicin). The patients were recruited in an outpatient oncology clinic (Alberto Cavalcanti Hospital, Belo Horizonte, Brazil), from 2015 to 2018. The exclusion criteria were as follows: a previous history of CT or radiotherapy; ventricular systolic dysfunction with a left ventricular ejection fraction (LVEF) of <50%, or patients in whom this condition could not be evaluated prior to CT; a history of heart disease or signs of decompensation (myocardial infarction, congestive heart failure, angina pectoris, or arrhythmia); valve disease (valve stenosis of any degree, regurgitation greater than mild severity) or uncontrolled arterial hypertension; liver or renal dysfunction; neurodegenerative diseases that required action by caregivers; and pregnant or breastfeeding women.

The criteria defined by López-Sendón et al. [18] were used to characterize cardiotoxicity: occurrence of new myocardial dysfunction (even asymptomatic), such as LVEF <50%, a 10% reduction in LVEF after the treatment, or an increase in troponin I (cTnI) or N-terminal type B natriuretic pro-peptide (NT-proBNP) levels after treatment.

### 2.2. Blood Samples and Measurements

Blood markers were evaluated at 3 time points: T0, pre-CT with doxorubicin; T1, post-CT (interval of up to 1 week after the end of doxorubicin administration); and T2, 1 year after the end of CT with doxorubicin and after fasting for 12 h. The RDW and CRP levels were obtained from medical records at these times. Morphological data were analyzed based on histological and immunohistochemical characteristics of the tumors. The cTnI and NT-proBNP levels were determined in serum samples by using chemiluminescence immunoassays in the Vitros 5600^®^ apparatus (OrthoClinical Diagnostics, Singapore).

### 2.3. Statistical Analyses

Categorical variables are presented as absolute and relative frequencies, and numeric variables are presented as the mean ± standard deviation or median (1st quartile–3rd quartile) for normally and non-normally distributed variables, respectively. To evaluate the significant differences between the three moments of the measurements, generalized estimating equation (GEE) models with a gamma distribution were used. Correlations between variables were assessed using Spearman’s correlation coefficient. Student’s *t*-test or the Mann–Whitney test was used to compare two independent groups, and analysis of variance (ANOVA) or the Kruskal–Wallis test with Bonferroni correction was used to compare more than two independent groups. The chi-square test was applied to compare frequencies between two or more groups. Analyses were performed using R software version 4.0.3 (R Foundation for Statistical Computing, Vienna, Austria), and a significance level of 5% was considered.

## 3. Results

Table 1 shows the demographic aspects of the population, comprising 80 patients with a mean age of 50.3 ± 12.7 years. There was a predominance of invasive ductal (90%) and human epidermal growth factor receptor 2 (HER2)-positive (43%) tumors. The mean dose of doxorubicin was 379 ± 62 mg. Thirty-three (41.2%) patients underwent treatment in combination with trastuzumab, and the mean length of time between the last doxorubicin cycle and the treatment with trastuzumab was 26 days (range 19–45 days)—that is, before T2. Cardiotoxicity occurred in 54 (67.5%) patients, but cardiotoxicity did not present a relationship with the molecular diagnosis of the tumor (*p* = 0.817). Four patients died before the T2 evaluation, due to causes unrelated to the treatment; for these patients, the values of the markers at T1 were used for T2.

**Figure 1:**
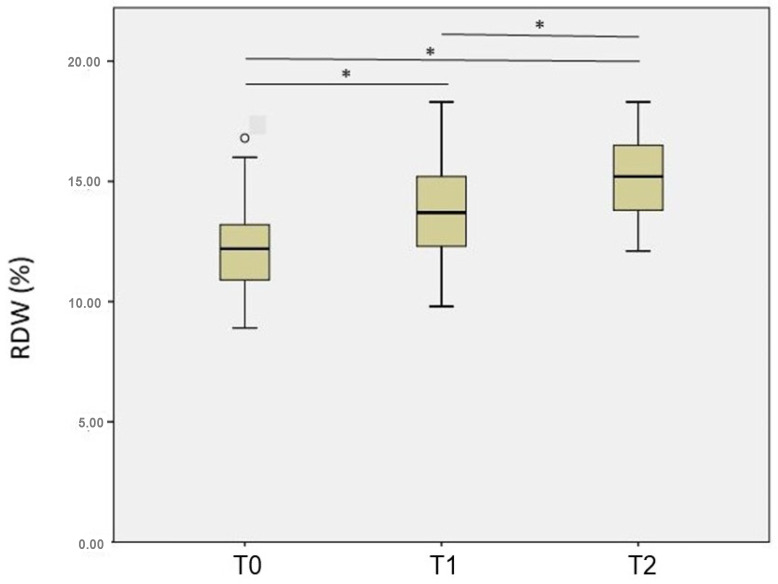
The RDW at T0 (before doxorubicin treatment), T1 (just after the treatment), and T2 (one year after the treatment) in women with breast cancer.* Significant: *p* < 0.05.

The RDW was significantly lower at T0 (12.0% ± 1.8%) compared with T1 (13.7% ± 2.2%) and T2 (14.7% ± 2.8%), and significantly lower at T1 compared with T2 (all *p* < 0.001) (Figure 1). The correlations between the RDW and the molecular diagnosis, HER2-positive status, and treatment with trastuzumab were not significantly different at any of the three analyzed time points (Table 2). In addition, the RDW was not different between the groups classified according to cardiotoxicity (Table 3).

CRP levels were lower at T2 (median 6.7 [5–10.1] mg/dL) compared with T0 (median 10.2 [5–18.6] mg/dL) (*p* = 0.033), and at T2 compared with T1 (median 9.3 [5–17.9] mg/dL) (*p* = 0.038). There was a significant and negative correlation between CRP and the RDW only at T1 (r = −0.344, *p* = 0.003). The CRP levels were not different between the cardiotoxicity and non-cardiotoxicity groups after doxorubicin treatment (*p* > 0.05). Only the cTnI and NT-proBNP levels changed during the follow-ups; these changes are part of the criteria for cardiotoxicity classification (Table 3).

## 4. Discussion

The RDW value was increased at the 1-year follow-up in women with breast cancer treated with doxorubicin, but it did not show a relationship with cardiotoxicity induced by the treatment. One year after chemotherapy, the RDW was higher than at the other time points, when all patients showed progression-free survival, which was measured from the date of definitive surgery after chemotherapy to T2 (the last follow-up). This finding suggests that the RDW could be a potential biomarker to evaluate the response to doxorubicin treatment. The variation in the size of erythrocytes, denoted by the RDW and obtained from complete blood cell count panels, is a reproducible, cost-effective, and accessible marker, and its inclusion as a CT-monitoring test would be desirable, due to the lack of a test that can assess the therapeutic response to doxorubicin [19].

The inflammatory process associated with cancer is a hallmark of tumor development and progression [20,21], and the RDW has been considered an inflammation-associated marker [20], with the potential to aid in determining the prognosis and overall mortality in these diseases, including several types of cancers [19]. An extensive inflammatory reaction is triggered, and leads to an increase in levels of circulating cytokines such as IL-6 and TNF-α [22] that can influence cell proliferation, survival, drug resistance, and migration of tumors [21]. The mechanism of the relationship between the RDW and the inflammatory process is still unknown, but it is believed that these cytokines suppress the maturation of erythrocytes and accelerate the entry of larger new reticulocytes into the peripheral circulation, thus increasing the RDW [22]. In addition, with regard to the association between the RDW and the nutritional status, malnutrition of patients due to iron, folate, and vitamin B12 deficiency, due to hyporexia caused by cancer, can affect hematopoiesis and, thus, amplify the heterogeneity in the red blood cell volume, leading to an increase in the RDW [22].

The contribution of the immune system to breast cancer prognosis has been investigated in several studies. Tumor-infiltrating lymphocytes (TIL) have been shown to provide prognostic and predictive values [23]. Curiously, triple-negative breast cancer is most likely to have tumors with >50% lymphocytic infiltrate, and there is a survival benefit from each 10% increase in TILs [24]. On the contrary, an increase in TILs is an adverse prognostic factor for survival in luminal HER2-negative breast cancer, suggesting a different biology of the immunological infiltrate in this subtype [25]. Additionally, Denker et al. [26] observed that the presence of tumor-associated lymphocytes in breast cancer was a predictor of response to anthracycline neoadjuvant chemotherapy. Unfortunately, TILs were not evaluated in the histological analyses carried out in the present study. Hence, further investigation regarding the relationship between TILs and the RDW is necessary to evaluate their potential as a marker of the local inflammatory process.

In a retrospective study including 395 patients that underwent surgical resection—breast-conserving surgery or total mastectomy, adjuvant chemotherapy, and radiotherapy—Lee et al. [27] observed that a lower RDW before treatment and then an increase in the RDW after treatment was significantly associated with poor survival. In addition, a high pretreatment RDW in patients with breast cancer was associated with poor overall and disease-free survival, along with a large tumor size, a high rate of lymph node metastases, tumor stage, advanced stage, a higher lymphocyte count, and high fibrinogen and CRP levels [28]. On the contrary, in this study, there was an increase in the RDW in patients after doxorubicin treatment.

The CRP levels decreased significantly over time, a finding in agreement with our previous study, which suggests that doxorubicin is capable of decreasing systemic inflammation [21]. CRP levels are used to detect inflammatory reactions and to help assess the progression of these diseases [29]; hence, the increased plasma concentrations are directly related to tumor burden and cancer prognosis [30]. Thus, the decrease in the CRP concentration between T0 and T2 represents a possible improvement in the inflammatory status after treatment. Curiously, the RDW was negatively correlated with CRP at T1. However, in chronic degenerative diseases, temporal variation can occur differently for markers and over a longer period; thus, there would not be an overlap of the results between the two variables [20]. Our results suggest that other mechanisms, in addition to inflammation, could contribute to stimulate reticulocyte release (represented by the RDW) in response to doxorubicin treatment, even one year after the treatment.

The use of doxorubicin causes numerous adverse events, the most common being spinal cord depression, bone loss, leukopenia, nausea, vomiting, anorexia, diarrhea, mucositis, alopecia, hemorrhagic cystitis, and, most importantly, cardiomyopathy [31]. The usual therapeutic regimen is the combination of doxorubicin with cyclophosphamide and taxanes (AC-T regimen). The treatment of the HER2-positive breast cancer also includes the association of the recombinant humanized monoclonal antibody, trastuzumab, which also has the potential to cause cardiotoxicity [32]. In our study, four (5%) patients died before T2, and 43% of the patients had the HER2-positive molecular diagnosis, which were then submitted to the treatment scheme with trastuzumab. However, the RDW was not different at the time points studied when comparing those who did and did not receive trastuzumab. There was no difference in the RDW according to the molecular or histological characteristics of the tumor. In contrast, Zou et al. [33] observed that the RDW was negatively correlated with the breast cancer histological grade and molecular typing in 653 patients.

The cTnI level is the gold standard in the diagnosis and prognosis of myocardial injury. It is usually increased after high doses of chemotherapy and is recognized as an independent biomarker of cardiotoxicity [34]. NT-proBNP originates from the cardiac ventricles, and is released into the circulation in response to ventricular volume expansion and pressure overload [35]. cTnI and NT-proBNP are important in the early detection of anthracycline-induced cardiotoxicity, associated with a ≥10% decline in LVEF [21,35,36]. In fact, cTnI and NT-proBNP levels at T1 and NT-proBNP levels at T2 were higher in the cardiotoxicity group, results that were expected because the elevation of these markers is part of the criteria to diagnose cardiotoxicity after doxorubicin treatment [18]. However, the RDW was not correlated with the cTnI and NT-proBNP levels. Our data suggest that the RDW is not a marker of anthracycline-induced cardiotoxicity, but it could be useful to track the therapeutic response, regardless of the tumor type.

Some limitations of this study should be considered. The main limitation is that the analysis was carried out in a single center and with a small number of patients. In addition, only patients with breast cancer treated with doxorubicin were included. Moreover, the number of studies that address the relationship between the RDW and cancer is still very small. There is a need for new studies with larger samples that corroborate the application of this index as routine in this condition. Finally, the mechanism that associates the increase in the RDW and the doxorubicin effect should be elucidated in additional studies.

## 5. Conclusions

This study suggests that RDW is not related to doxorubicin-induced cardiotoxicity, but it could be a widely accessible and inexpensive marker to evaluate the response to this anthracycline in women with breast cancer.

## Figures and Tables

**Table 1:** Descriptive analysis of patients with breast cancer treated with doxorubicin.

Variable	Patients (*n* = 80)
Mean age	50.3 ± 12.7 years
Histological diagnosis—*n* (%)	
Invasive ductal carcinoma	72 (90%)
Invasive lobular carcinoma	6 (7%)
Special types	2 (8%)
Molecular diagnosis—*n* (%)	
Luminal	25 (32%)
HER2	34 (43%)
Triple-negative	20 (25%)
ER—*n* (%)	
Positive	39 (49%)
Negative	40 (51%)
PR—*n* (%)	
Positive	28 (35%)
Negative	51 (65%)
Dose DOXO	379 ± 62 mg/m^2^
Trastuzumab deruxtecan—*n* (%)	
Yes	33 (41%)
No	47 (59%)
RT Post-CT—*n* (%)	
Yes	53 (66%)
No	27 (34%)
Death—*n* (%)	
Yes	4 (5%)
No	76 (95%)
Cardiotoxicity—*n* (%)	
Yes	54 (68%)
No	26 (32%)

Abbreviations: DOXO–doxorubicin; HER2—human epidermal growth factor receptor 2; ER—estrogen receptor; PR—progesterone receptor; RT Post-CT—radiotherapy post-chemotherapy.

**Table 2:** Comparison of RDW with molecular diagnosis and use of trastuzumab at different time points.

	RDW		
	T0	T1	T2
Molecular diagnosis			
Luminal	12.0 (11.0—12.8)	14.5 (12.4—16.7)	14.6 (13.6—16.7)
HER2	12.4 (11.1—14.1)	13.4 (12.3—14.1)	14.8 (13.8—16.1)
Triple-negative	11.0 (9.8—12.3)	13.9 (12.4—15.9)	15.5 (12.4—16.1)
*p*-Value ^K^	0.126	0.176	0.708
Trastuzumab use			
Yes	12.4 (11.1—13.9)	13.2 (12.3—14.1)	14.8 (14.1—16.4)
No	11.8 (10.7—12.8)	14.1 (12.4—15.9)	14.6 (13.4—16.6)
*p*-Value ^M^	0.167	0.058	0.688

^K^ Kruskal–Wallis test; ^M^ Mann–Whitney test. Abbreviations: T0—initial time; T1—time just after chemotherapy treatment; T2—time one year after completion of chemotherapy; RDW—red cell distribution width; HER2—human epidermal growth factor receptor 2. Significant: *p* < 0.05.

**Table 3:** Comparison of RDW and cardiac markers with cardiotoxicity in breast cancer patients treated with doxorubicin.

	Cardiotoxicity		
	Yes (*n* = 54)	No (*n* = 26)	*p*-Value ^M^
Initial Time (T0)			
RDW index	12.0 (10.8—12.8)	12.7 (10.9—14.2)	0.178
CRP	10.2 (5.0—22.5)	10.4 (5.7—15.6)	0.699
cTnI	0.012 (0.012—0.012)	0.012 (0.012—0.012)	0.557
NT-proBNP	52.4 (34.8—85.3)	63.3 (49.0—86.7)	0.223
Per CT (T1)			
RDW	13.2 (12.3—14.6)	14.3 (13.2—15.3)	0.129
CRP	9.7 (5.0—17.9)	7.2 (5.0—12.9)	0.491
cTnI	0.017 (0.012—0.028)	0.012 (0.012—0.013)	0.002 *
NT-proBNP	77.2 (45.1—162.0)	42.1 (32.8—53.5)	0.001 *
One year Post-CT (T2)			
RDW	15.2 (13.6—16.5)	14.2 (13.5—15.7)	0.197
CRP	6.4 (5.0—12.5)	8.2 (6.0—10.1)	0.288
cTnI	0.012 (0.012—0.018)	0.012 (0.012—0.012)	0.308
NT-proBNP	80.3 (38.0—147.0)	48.7 (32.8—66.3)	0.011 *

^M^ Mann–Whitney U test. Abbreviations: RDW—red cell distribution width index; CT—chemotherapy; cTnI—cardiac troponin I (ng/mL); NT-proBNP—N-terminal-prohormone brain natriuretic peptide (or N-terminal of the B-type natriuretic peptide) (pg/mL).* Significant: *p* < 0.05.

## Data Availability

The data that support the findings of this study are available on request from the corresponding author, K.B.G.
